# Locomotion Decoding (*LocoD*): An Open‐Source and Modular Platform for Researching Control Algorithms for Lower Limb Assistive Devices

**DOI:** 10.1155/abb/3160186

**Published:** 2026-01-04

**Authors:** Bahareh Ahkami, Kirstin Ahmed, Morten B. Kristoffersen, Max Ortiz-Catalan, Andrea Tigrini

**Affiliations:** ^1^ Center for Bionics and Pain Research, Gothenburg, Sweden; ^2^ Department of Electrical Engineering, Chalmers University of Technology, Gothenburg, Sweden, chalmers.se; ^3^ Department of Orthopedic Traumatological Pathology, Rizzolli Orthopaedic Institute, Bologna, Italy; ^4^ Department of Mechatronic, Electrical Energy and Dynamic Systems, UCLouvain, Ottignies-Louvain-la-Neuve, Belgium; ^5^ The BioRobotics Institute, Scuola Superiore Sant’Anna, Pisa, Italy, sssup.it; ^6^ Department of Engineering Technology, Technical University of Denmark, Copenhagen, Denmark, dtu.dk; ^7^ Department of Orthopaedics, Institute of Clinical Sciences, Sahlgrenska Academy, University of Gothenburg, Gothenburg, Sweden, gu.se; ^8^ Prometei Pain Rehabilitation Center, Vinnytsia, Ukraine; ^9^ Center for Complex Endoprosthetics, Osseointegration, and Bionics, Kyiv, Ukraine

**Keywords:** biomedical signal processing, electromyogram, lower limb prosthetic control, open-source software, prostheses

## Abstract

**Background and Objective:**

Commercially available motorized prosthetic legs use exclusively nonbiological signals to control movements, such as those provided by load cells, pressure sensors, and inertial measurement units (IMUs). Although the use of biological signals of neuromuscular origin can provide more natural control of leg prostheses, these signals cannot yet be captured and decoded reliably enough to be used in daily life. Indeed, decoding motor intention from bioelectric signals obtained from the residual limb holds great potential, and therefore, the study of decoding algorithms has increased in the past years, with standardized methods lacking.

**Methods:**

In the absence of shared tools to record and process lower limb bioelectric signals, such as electromyography (EMG), we developed an open‐source software platform to unify the recording and processing (preprocessing, feature extraction, and classification) of EMG and nonbiological signals amongst researchers with the goal of investigating and benchmarking control algorithms. We validated our locomotion decoding (LocoD) software by comparing the accuracy in the classification of locomotion mode using three different combinations of sensors (1 = IMU + pressure sensor + EMG, 2 = EMG, 3 = IMU + pressure sensor). EMG and nonbiological signals (from the IMU and pressure sensor) were recorded while able‐bodied participants (*n* = 21) walked on different surfaces, such as stairs and ramps, and this data set is also released publicly along with this publication. *LocoD* was used for all recording, preprocessing, feature extraction, and classification of the recorded signals. We tested the statistical hypothesis that there was a difference in predicted locomotion mode accuracy between sensor combinations using the Wilcoxon signed‐rank test.

**Results:**

We found that the sensor combination 1 (IMU + pressure sensor + EMG) led to significantly more accurate and improved locomotion mode prediction (Accuracy = 93.4 ± 3.9) than using EMG (Accuracy = 74.56 ± 5.8) or IMU + pressure sensor alone (Accuracy = 90.77 ± 4.6) with *p*‐value <0.001.

**Conclusions:**

In this study, we introduced and validated the functionality of *LocoD* as an open‐source and modular platform to research control algorithms for prosthetic legs that incorporate bioelectric signals.

## 1. Introduction

After amputation, most people seek independence and reintegration into previous activities and often acquire a prosthetic limb as part of this effort. Users report wanting their prosthetic limb to be versatile and stable to support them in activities like sitting to standing up, and that they can feel safe and confident using it [1]. The field of lower limb prostheses has witnessed a trend toward developing more motorized prosthetic legs. Users who previously felt unable to live as independently as they wished are now able to rely on their motorized prostheses to aid them in everyday living [2, 3]. Recent advancements in battery technology and lightweight materials have further enhanced the usability and comfort of these devices, making them even more practical and appealing for daily use. Additionally, the introduction of open‐source prosthetic legs (OSL) represents a significant breakthrough, offering customizable and accessible solutions that can be tailored to individual needs and advancing research in the field [2]. Nonetheless, the state‐of‐the‐art motorized lower limb prosthesis available to the public utilize nonbiological sensors such as load cells, pressure sensors, and inertial measurement units (IMUs) to detect the current locomotion mode and its transitions [4]. These devices base their control algorithms on mechanical interactions of the user with the environment, rather than the user’s ambulatory intents. This forces the user to use the prosthesis in an unnatural way, dissimilar to how they would use their intact limb. Overall, lower limb prostheses are still far from providing users with a natural gait, and there is a palpable need to develop better and more natural control systems for prosthetic legs [4–6].

Several comprehensive reviews have surveyed advances in control algorithms for lower limb prostheses, focusing on neuromechanical modeling, sensor fusion, and machine learning strategies. For example, Fluit et al. [7] compared control strategies in commercial and research knee prostheses, highlighting limitations in adaptability and responsiveness. Tucker et al. [8] provided a comprehensive overview of control strategies for active prosthetics and orthotics, emphasizing the role of user intent recognition. Voloshina and Collins [9] discussed the trade‐offs in design and control approaches for achieving both biomechanical realism and real‐time control in lower‐limb devices. These works, among others, underscore the ongoing efforts to refine control systems for improved user performance [4, 10–12].

One major avenue of development has been the integration of electromyography (EMG) signals. Prior work has shown that fusing EMG with nonbiological sensors can enhance classification accuracy in predicting locomotion modes. For instance, Huang et al. demonstrated improved transition detection using neuromuscular‐mechanical fusion [13], while Spanias et al. [14] reported gains in pattern recognition performance through the addition of mechanical sensors to EMG‐based systems. Similarly, Simon et al. and Krausz et al. developed hybrid systems that leveraged both physiological and environmental inputs [15, 16]. These studies support the hypothesis that EMG, when combined with inertial or pressure data, improves the robustness and responsiveness of control algorithms in prosthetic applications.

Despite this obvious control gain, there are still no commercial devices that use EMG signals in the prosthetic leg market. Furthermore, the variability in study designs and datasets, as well as proprietary software platforms for recording and processing EMG signals that are not publicly available, hinders comparisons between control algorithms and may be limiting the advancement in this field. This lack of standardization across research tools and algorithms remains a significant barrier to advancing lower limb prosthetic control.

One example of a proprietary software solution is the Control Algorithms for Prosthetics System (CAPS), developed at the Shirley Ryan AbilityLab, Chicago, USA [17, 18]. Similarly, the Joint Department of Biomedical Engineering at North Carolina State University & University of North Carolina at Chapel Hill, USA, has developed a software which records and processes data recorded from EMG and nonbiological sensors for real‐time control of upper and lower limb prosthetics [18, 19]. These platforms have been used to produce ground‐breaking research; however, due to their proprietary nature, it is not possible to openly access or modify them, preventing them from being used as common research tools. One existing, open‐source software solution for upper limb prosthetics is *BioPatRec* [20], which is suitable for collegial development of upper limb control systems. However, a limitation with *BioPatRec* being used for lower limb prosthetic control is that the quasi‐cyclic nature of walking poses different processing requirements compared to upper limb dynamics.

In this work, our goal was to advance the field of lower limb prosthetic control by developing an open‐source and modular research platform for locomotion decoding (LocoD). LocoD aims to serve as a unified foundation for research, allowing for seamless comparisons of various processing and control algorithms. In this study, we utilized LocoD to evaluate different combinations of biological and nonbiological sensors, assessing their accuracy in detecting locomotion during gait. Our findings demonstrate that LocoD can be used to conduct research in lower limb prosthetic control and thereby potentially accelerate the development of technical solutions to close the functionality gaps in lower limb prosthetics.

A preprint of this study was previously made publicly available via SSRN [21], and this article represents the final version following peer review.

## 2. Methods

### 2.1. LocoD Software Platform and Data Repository

*LocoD* was developed using MATLAB (2021b, MathWorks, USA) [22], incorporating a user interface inspired by *BioPatRec* [20]. *LocoD*’s main release branch is maintained on GitHub, which also includes *LocoD* build documentation, sample use cases, and user manuals [23].

### 2.2. Signal Acquisition and Processing (Preprocessing, Feature Extraction, and Classification)

LocoD can acquire signals in real time using supported hardware (e.g., DELSYS Trigno system (Trigno, Delsys, USA) [24]) or process prerecorded datasets, provided they follow LocoD’s data structure. Inputs may include biological and/or mechanical signals such as EMG, IMU, pressure sensors, or load cells. The required structure specifies both the organization of the data (MATLAB files with predefined channel order) and the naming conventions for each parameter.

The ultimate output from *LocoD* is a prediction of the locomotion mode, albeit outputs at each module in the signal chain can also be obtained. In each recording session, the operator should:

A. Select the signal recording settings, for example, the combination of sensors used and the type of data acquisition system.
B. Start signal acquisition while the participant performs a predefined set of movements (e.g., walking or climbing stairs). The actual locomotion mode should be keyed in while the recording is ongoing. This will be used as the classifier’s label input. The operator then terminates the recording session.

Then processing consists of signals conditioning and decoding, and the user must:

A. Specify the preprocessing and processing algorithms and their parameters, including, for example, the type of features to be extracted, how the signal should be divided into windows, and how cross‐validation takes place.
B. Train the classifier and obtain the classification accuracy, which can then be compared between different sensor combinations or different methods of classification.

### 2.3. Software Architecture

*LocoD* uses the object‐oriented software development paradigm. It employs MATLAB classes to define various concepts. The use of classes allows changes, enhancements, or replacements in any part of the program, independent of other modules or classes. Different graphical user interfaces (GUIs) allow researchers to change and test different parameters related to every step of recording and signal processing (preprocessing, feature extraction, and classification).

**Figure 1:**
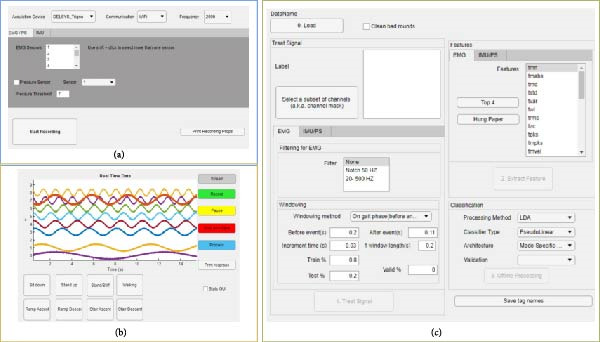
LocoD graphic user interface (GUI). (a) Analog front‐end GUI to set recording properties, (b) recording session GUI to observe signal and keyed‐in tags in locomotion mode, and (c) offline processing GUI to process signal from preprocessing to validation.

Developers can also modify or add any parameters to the GUI (Figure 1).

**Figure 2:**
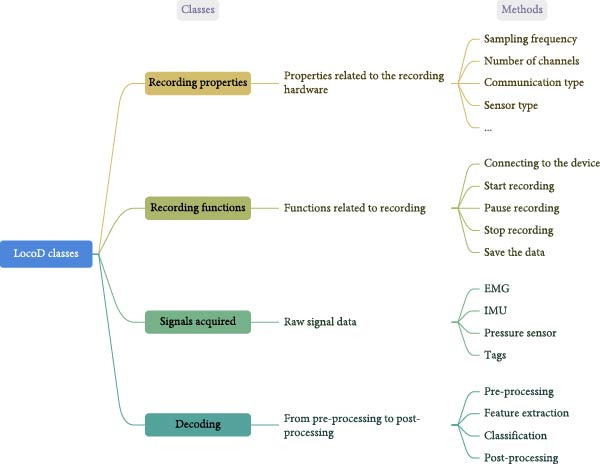
Different classes of LocoD and their role in recording or processing signals. EMG, electromyogram; IMU, inertial measurement unit.

The software architecture of *LocoD* can be thought to consist of four classes: (1) recording properties; (2) recording functions; (3) signals acquired; and (4) decoding (Figure 2).

The *Recording Properties* class consists of all settings to record and store signals, for example, number and order of channels, data acquisition device, sampling frequency, and type of sensors. There is a dedicated GUI where these settings can be changed (Figure 1a).

Objects of the *Recording functions* class facilitate connection to the data acquisition device, streaming the signals, pausing or stopping streaming, and saving the recorded signal in a file for further processing (Figure 1b).

The *Signals acquired* class stores recorded signal together with its complementary metadata, such as sampling frequency, number of channels, and recording device. An instance of a signal is a matrix of sample values for each channel (EMG, IMU, pressure sensor, or any additional sensors). In addition, locomotion modes (tags) indicated by the operator are stored here.

The *Decoding* class handles all processing steps (Figure 2). Processing is performed after the signal is recorded and consists of three major steps: (1) preprocessing; (2) feature extraction, and (3) classification. The “offline classification GUI window” provided in *LocoD* allows the operator to configure and run the process (Figure 1c).

**Figure 3:**
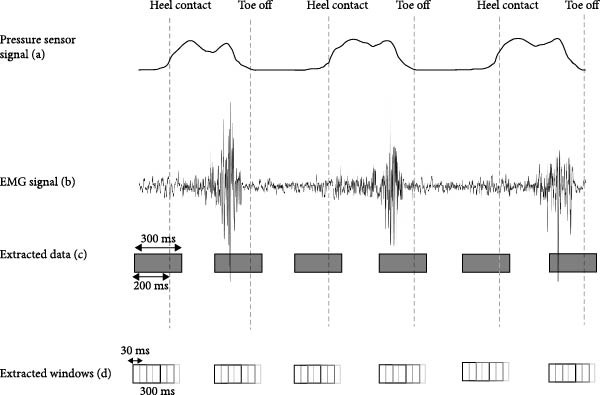
(a) Pressure sensor signal, (b) EMG signal, (c) 300 ms of extracted data around each gait phase, including 200 ms from before each gait phase and 100 ms from after each gait phase, and (d) extracted windows with 30 ms of increment from extracted data. The same overlapping windowing scheme was applied to EMG, IMU, and pressure sensor signals to ensure consistent feature extraction across modalities.

*Step 1* (*Preprocessing*): Preprocessing consists of two parts of filtering and windowing. Raw data cannot be used directly for classification; therefore, preprocessing maximizes information from the recorded signal by filtering the signal and removing noisy channels. Next, the pressure sensor signal is used to indicate heel contact and toe‐off gait events, for which the surrounding signal time series is taken and cut into small windows. Signal time span around the gait events can be adjusted as well as the windowing method (incremental or nonincremental) (Figure 3). Then, each signal window gets a label based on the locomotion transition tags, which were previously keyed in by the operator for each transition. The operator was instructed to key in each tag for a new locomotion mode right before the participant entered it. The manually entered tags serve only as ground truth for validation, without influencing the recognition process itself.

**Figure 4:**
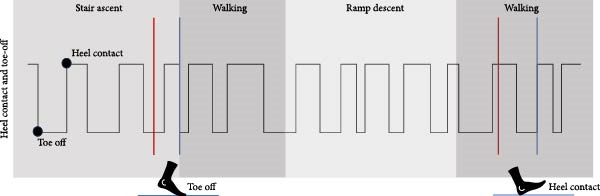
The red line is the tag entered by the operator. For the transition of walking to stair ascent and stair ascent to walking, the transition tags are applied to the next toe‐off onwards (blue line). In the case of ramp descent to walking and stair descent to walking, they are projected to the next heel contact moment (blue line).

To eliminate human timing error with the keyed‐in tags, entered tags are automatically projected to the next heel contact or toe‐off moment (heel contact and toe‐off moments extracted from the pressure sensor). For the transition of walking to stair ascent and stair ascent to walking, the transition tags are applied to the next toe‐off onwards, while in the case of ramp descent to walking and stair descent to walking, they are projected to the next heel contact moment [25] (Figure 4). In all other cases, labels were aligned to the next gait event (toe‐off or heel strike).

Based on the sequence of previously entered tags and the current ones, the system determines the current locomotion state and the state to which the participant is transitioning. This allows the system to automatically mark and save the signal windows surrounding these gait events under the correct transition class, without requiring any further input from the operator.

**Figure 5:**
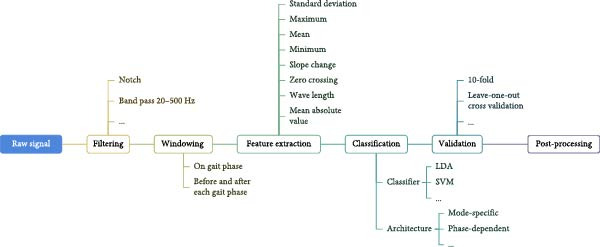
Steps of processing raw signal to train and validate the classifiers detecting locomotion mode.

*Step 2* (*Feature extraction*): Common classifiers do not use time series as input to classify different classes. Instead, features extracted from time windows are fed as input (Figure 5). These features can be extracted in the time domain, for example, mean absolute value and standard deviation, or in the frequency domain, such as power spectral density, or in the time–frequency domain, such as wavelet [26]. *LocoD* was released with 20 different features in the time and frequency domains. This is based on the feature extraction method that was implemented in *BioPatRec* [20].

**Figure 6:**
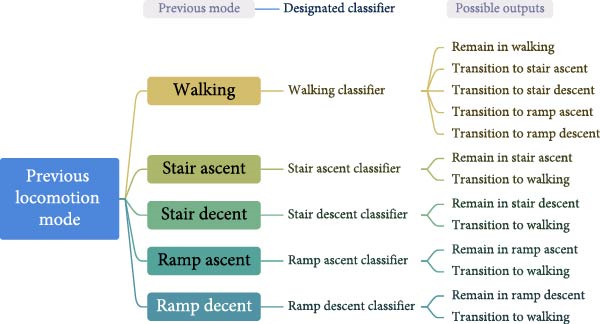
Mode‐specific classification architecture. There is one classifier for each locomotion mode. The classifier is chosen based on the previous mode. Each classifier has limited classes, for example, if the previous class is stair ascent, the stair ascent classifier will be used. In this classifier, the output can remain in stair ascent or transition to walking.

*Step 3* (*Classification and validation*): Extracted features get passed to a classifier that detects the locomotion mode. The classifier’s architecture used with *LocoD* has two characteristics: mode‐specific (Figure 6) and phase‐dependent. Classification is performed for features of signal windows extracted around any gait phase change, heel contact, and toe‐off (phase‐dependent). Here, based on the label of the previous window, we can limit the possible labels for the current window. For example, if the previous window was classified as a stair/ramp ascent/descent locomotion mode, then the current window can only be classified as the same or walking (hence mode‐specific) [27]. Therefore, *LocoD* employs an array of classifiers that are chosen based on the prediction outcome of the previous window. Considering having five locomotion modes and two gait phases, this leads to 10 classifiers. The classifier used for the validation in this study is based on linear discriminant analysis (LDA), but it is also possible to use support vector machine (SVM) classifiers with this release of *LocoD*.

After classification, there is an optional step of cross‐validation. *LocoD* has built‐in n‐fold cross‐validation. To address the imbalance between the number of steady‐state samples and transition samples in these types of datasets, we recommend using this validation feature. This approach ensures a more accurate and robust evaluation of the classifier’s performance across different locomotion modes. The outcome measure is calculated as the classification accuracy of different locomotion modes in two conditions, steady‐state and transition. Here, steady‐state accuracy is the percentage of windows correctly classified when there was no transition, while transitional accuracy is defined as the percentage of windows accurately classified from one locomotion mode to the other (e.g., transition from walking to stair ascent).

#### 2.3.1. Data Collection and Repository

**Figure 7:**
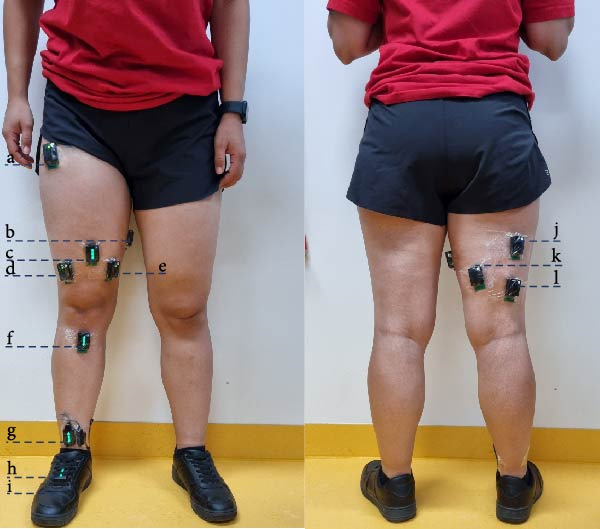
Sensor placement on the front and rear of a participant includes the following: (a) tensor fasciae latae, (b) gracilis, (c) rectus femoris and IMU 1, (d) vastus lateralis, (e) vastus medialis, (f) IMU 2, (g) pressure sensor adapter, (h) IMU 3, (i) pressure sensor in the insole, (j) semitendinosus, (k) biceps femoris long head, and (l) biceps femoris short head.

A data repository recorded using *LocoD* is included in this release [28] and consists of sample data from 12 female and nine male able‐bodied subjects with no prior LocoD experience. The participants were between 23 and 31 years old, with a mean age of 27 ± 2.3 years. The recorded data corresponds to one recording per participant, digitalized at 2 kHz. Data were collected using 10 Delsys Trigno sensors [24], each capable of recording both EMG and IMU signals. Of these 10 sensors, eight were used for EMG recordings. They were placed on the semitendinosus, biceps femoris long and short heads, tensor fasciae latae, rectus femoris, vastus lateralis, vastus medialis, and gracilis muscle [4], followed SENIAM guidelines [29]. The rectus femoris sensor also provided IMU data in addition to EMG. Two further Trigno sensors were mounted below the knee and on the foot, where EMG channels were disabled and only IMU data were collected. This configuration provided IMU signals from three sites: above the knee (rectus femoris), below the knee, and the foot. A pressure sensor integrated into an insole was used to capture heel strike and toe‐off events (Figure 7).

**Figure 8:**
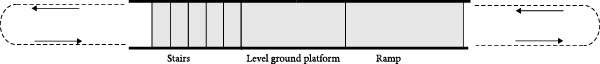
Data recording circuit: Stairs, level ground walking, and ramp.

Data consists of 30 trials of a circuit per participant with sensors on their leg, which included walking, stair ascent, stair descent, ramp ascent, and ramp descent (Figure 8). These surfaces were selected as they are the most common movements in daily life. All data were collected under identical conditions at Sahlgrenska University Hospital Mölndal, Sweden. To ensure consistent conditions for all participants, the environment was carefully controlled. The setup comprised 200 cm of walking, a ramp with a 7° slope, 140 cm of level ground walking, a staircase with six steps (each with a 30 cm tread and a 10 cm riser), followed by another 200 cm of walking.

For this study, written informed consent from participants and ethical approval from the Swedish Ethical Review Authority (2020‐06479) were obtained.

### 2.4. Validation of LocoD

To validate *LocoD*, we processed the recordings in the data repository in three different sensor combinations: combined nonbiological signals + EMG, EMG only, and nonbiological signals (IMU + pressure sensor) only. EMG data were filtered using a 20‐500 Hz bandpass filter [30] and a sixth‐order notch filter at 50 Hz to remove power line interference. Signal blocks were extracted around each gait phase (heel contact and toe‐off) from 200 ms before to 100 ms after (300 ms in total). Windows of 200 ms with 30 ms increments were then extracted from this data (Figure 3). Mean absolute value, waveform length, number of zero crossings, and slope sign change were extracted from each window of the EMG signal [31, 32], whereas mean, maximum, minimum, and standard deviation features were extracted from each window of IMU and pressure sensors [27, 33].

Extracted features of selected sensor channels were merged into the feature vectors used for classification. For example, for the case of nonbiological signals + EMG we concatenated features from the IMU channels and pressure sensor with the features from EMG channels in the same feature vector. We had three separate IMU sensors, each IMU had six axes (three for gyroscope, and three for accelerometer), and from each axis we extracted four features, which resulted in 72 features in total. We had one channel of a pressure sensor, from which we extracted four features. We had eight channels of EMG signals from which we extracted four features each (32 features in total). Putting all features together, a total of 108 features were extracted per time window. Extracted features were passed to an LDA classifier with a phase‐dependent mode‐specific architecture and 10‐fold cross‐validation [27]. The classifier was trained per participant.

Accuracy of classification is reported in three ways: considering all windows, only locomotion transition windows, and only steady‐state windows (when no transition is happening).

Accuracy is emphasized in this study because it is the most widely recognized and utilized outcome measure for controlling lower limb prosthetics through EMG and machine learning methods [4–6].

### 2.5. Statistical Analyses

To examine whether adding EMG to the nonbiological signals has a significant impact on classification accuracy, we compared the classification results of using nonbiological data alone versus using nonbiological signals + EMG and EMG data alone versus nonbiological signals + EMG data for each participant. Given the nonnormality of our data, verified by the Kolmogorov–Smirnov test, and the pairwise nature of our comparison, we opted for the Wilcoxon signed‐rank test (MATLAB 2021b, USA).

## 3. Results

The locomotion detection algorithm was tested with the participation of 21 able‐bodied individuals. Table 1 provides an overview of the classification accuracy for each locomotion mode, considering various sensor combinations. These accuracy values are averaged across all participants.

**Table 1:** Classification accuracy of each locomotion mode and transitions in three different sensor combinations (nonbiological signals + EMG ^∗^, EMG, nonbiological signals) averaged over all participants.

Locomotion modes	Nonbiological signals + EMG	EMG	Nonbiological signals
Walking	96.38	95.27	95.73
Stair ascent	93.27	86.30	91
Stair descent	95.88	82.54	92.60
Ramp ascent	97.70	93.31	96.31
Ramp descent	97.94	93.02	96.73
Walk to stair ascent	90.61	68.68	85.79
Walk to stair descent	89.85	60.02	86.38
Walk to ramp ascent	92.04	63.18	86.01
Walk to ramp descent	86.79	38.47	82.05
Stair ascent to walk	94.30	88.52	90.86
Stair descent to walk	95.75	84.32	94.10
Ramp ascent to walk	92.42	65.89	89.01
Ramp descent to walk	91.73	66.25	88.87

Abbreviation: EMG, electromyogram.

**Figure 9:**
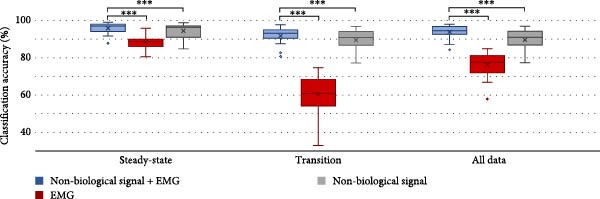
Classification accuracy in three different sensor combinations (nonbiological signals + EMG, EMG, nonbiological signals). Average over all participants (1) steady‐state locomotion mode (While there was no transition between different surfaces), (2) transition between different locomotion modes (e.g., transition from stair ascent to level ground walking), and (3) all data refer to the combined accuracy across both steady‐state and transition windows. The Y‐axis ranges from 30% to 100% for clearer presentation. A cross in the middle of each box plot represents the mean.  ^∗∗∗^ indicates a *p*‐value <0.001.

Figure 9 shows classification accuracy in three different sensor combinations (nonbiological signals + EMG, EMG, nonbiological signals). Average over all participants in the three scenarios of steady‐state, transition from one locomotion to another mode, and a combination of both steady‐state and transition data together.

## 4. Discussion

In this study, we introduced *LocoD*, open‐source platform designed to enable the seamless development and evaluation of algorithms for controlling prosthetic legs using a variety of signal sources. Our objective was to test this platform and evaluate the locomotion detection accuracy when using different sensors, such as IMU + pressure sensor (nonbiological signals), EMG, and IMU + pressure sensor + EMG. In this article, we did not aim to present a novel prosthetic control system, but to introduce LocoD as a tool that helps researchers to investigate such systems. Although the cyclic nature of gait can facilitate locomotion intention recognition, it also poses unique requirements such as event‐driven segmentation, phase‐dependent classification, and transition handling. Software that was originally developed for upper‐limb prosthetic control does not natively support these features. LocoD was therefore designed to integrate these modules, making it suitable for lower‐limb applications.

We validated *LocoD* by showing that the addition of EMG to nonbiological sensors, such as IMU and pressure sensors, significantly enhances locomotion detection classification accuracy (*p*‐value <0.001). Our findings are consistent with the existing literature [4, 5, 13, 14] and reiterate the value of EMG as an information source for improving prosthetic leg control. Regarding previous work in the field [4, 5, 13, 14], a distinguishing finding of our study lies in the accuracy of individual movements. For example, the accuracy of walking to ramp descent was drastically lower than the other movements (Table 1), whereas no such variance between movements has been reported in the literature. This divergence could be attributed to differences in the experimental setup and the unique execution of movements by individuals with amputation and those with able bodies, or likely due to the greater biomechanical variability of these movements. Descent involves eccentric muscle activity and diverse strategies for stability, resulting in more variable EMG and kinematic patterns compared to ascent or level walking [34]. Future work could address this reduced robustness by incorporating adaptive classification techniques, which may capture the continuous nature of biomechanical modulation during descent. Expanding the training dataset with larger samples of descent tasks, or including features sensitive to eccentric muscle activity, may also improve decoding accuracy [35].

One can argue that in lower limb prosthetic control, improvements that might appear incremental can be of substantial importance, particularly in mitigating the risk of adverse events such as falls. However, a critical evaluation of the translation of our findings into clinically meaningful outcomes is essential. As a next step, real‐time testing involving participants with amputations and active prosthetic legs is imperative to validate the practical applicability of our results. This aligns with findings by Hargrove et al. [18], who emphasized the necessity of real‐world testing to confirm the laboratory results.

Our study had several limitations, such as the reliance on able‐bodied participants for validation. This choice was intentional, as we wanted to thoroughly test the platform and its algorithms in a controlled environment before advancing to trials with participants with amputations. This approach aligns with common practices in developing new algorithms, where initial testing on able‐bodied individuals is standard for safety reasons [36]. Nevertheless, it does not fully capture the performance expected and the challenges faced by people with amputation. Although this study used a full set of sensors in able‐bodied participants, LocoD’s modular framework enables systematic evaluation of reduced or alternative sensor configurations, making it adaptable to different populations. Additionally, our platform primarily focuses on continuous locomotion detection, which may not be as effective in noncontinuous or nonweight‐bearing movements, such as sitting or standing up. The integration of EMG signals also presents challenges, requiring precise sensor placement and calibration, which can be time‐consuming and may vary between users, impacting data consistency.

Future steps for LocoD include establishing communication with the Open Source Leg (OSL) or other prosthetic legs [2], implementing more advanced control techniques such as deep neural networks, and expanding the sensor repertoire to encompass various biological and nonbiological sources, including EEG and load cells. In addition, regression‐based modules represent an important direction for future development (e.g., estimating ground reaction forces from proximal sensors and EMG [37]), which could further improve robustness. Other promising avenues include advanced postprocessing techniques to attenuate misclassifications and source‐selection methods to filter out less informative signals. In this study, pressure sensors were used to detect heel strike and toe‐off events due to their robustness and reliability in providing ground truth. However, IMUs can also serve this purpose, and LocoD’s modular framework allows for the integration of such. Another avenue for improvement involves optimizing the window size used in data analysis. Most studies, including ours, have relied on a fixed window size. Developing a metric that adapts the window size to walking speed could lead to more personalized and effective control. Finally, an automatic labeling system represents a logical next step, reducing operator dependance and eliminating variability in manual annotation.

By addressing these challenges through ongoing research and development, we hope that LocoD will help researchers to bring their prosthetic control systems closer to successful deployment in clinical practice, ultimately enhancing the quality of life for users of prosthetic devices.

The LocoD platform offers significant potential for applications in the realm of lower limb assistive devices. By integrating EMG signals with IMUs and pressure sensors, LocoD can significantly enhance the natural control and responsiveness of motorized prosthetic limbs. Looking ahead, this technology can be adapted to power exoskeletons and orthotic devices, enabling smoother transitions and more intuitive movements for individuals with lower limb neuromuscular impairments. Moreover, we hope for LocoD to serve as a collaborative research platform, fostering innovation and ultimately driving advancements in the field of lower limb prosthetics and orthotics.

## 5. Conclusion

Restoration of lower limb function in the real world is the ultimate goal in the field of bionic lower limbs, and natural control is an important part of it. None of the commercial prosthetic legs in the market use EMG as their control input, even though published results indicate that its use, in combination with other nonbiological sensors, can improve control. Our results support previous reports showing that EMG can be a useful source of information for the natural control of a prosthetic leg.

*LocoD* was developed as an open‐source and modular platform specifically to address the need for a shared research tool in this area. By making LocoD open‐source, we aim to encourage the sharing and comparison of algorithms on a common platform, ultimately accelerating research and development in prosthetic leg control. The platform’s ability to integrate various sensors and process both biological and nonbiological signals provides a versatile foundation for researchers to develop and test new control algorithms. As research continues, we hope for LocoD to play a vital role in advancing the field toward prosthetic legs that are seamlessly and naturally controlled by their users.

## Ethics Statement

This study was conducted in accordance with the ethical guidelines outlined by the Sweden Ethics Committee, which approved the experimental protocol (Approval Number 20‐06479). Prior to participation, all individuals received a comprehensive explanation of the study’s objectives and procedures. Informed consent was obtained from all participants through signed consent forms. In addition to consenting to participate in the study, all individuals also provided written consent for the publication of any images captured during the course of the research.

## Disclosure

All authors edited and approved the final manuscript.

## Conflicts of Interest

Max Ortiz‐Catalan and Morten B. Kristoffersen have consulted for Integrum AB. However, their affiliation with Integrum AB did not influence the design, execution, analysis, or interpretation of the results presented in this study. Bahareh Ahkami and Kirstin Ahmed declare no competing interests.

## Author Contributions

Max Ortiz‐Catalan conceptualized the platform. Bahareh Ahkami designed and programed the platform. All authors designed the study. Morten B. Kristoffersen supervised the implementation of the platform and assisted with platform testing. Max Ortiz‐Catalan and Kirstin Ahmed supervised the project. Max Ortiz‐Catalan secured funding. Bahareh Ahkami and Kirstin Ahmed drafted the manuscript.

## Funding

This work was supported by the Promobilia Foundation, the IngaBritt and Arne Lundbergs Foundation, and the Swedish Research Council (Vetenskapsrådet).

## Data Availability

The datasets gathered and analyzed during the current study are available in the Zenodo repository at https://zenodo.org/records/7534679. The software developed for this study is publicly accessible on GitHub at https://github.com/biopatrec/LocoD.
